# Assessment of the Massachusetts Flexible Services Program to Address Food and Housing Insecurity in a Medicaid Accountable Care Organization

**DOI:** 10.1001/jamahealthforum.2023.1191

**Published:** 2023-06-02

**Authors:** Jessica L. McCurley, Vicki Fung, Douglas E. Levy, Sydney McGovern, Christine Vogeli, Cheryl R. Clark, Stephen Bartels, Anne N. Thorndike

**Affiliations:** 1 Division of General Internal Medicine, Massachusetts General Hospital, Boston; 2 Harvard Medical School, Boston, Massachusetts; 3 Department of Psychology, San Diego State University, San Diego, California; 4 Mongan Institute Health Policy Research Center, Massachusetts General Hospital, Boston; 5 Division of General Internal Medicine & Primary Care, Brigham and Women’s Hospital, Boston, Massachusetts

## Abstract

**Importance:**

Health systems are increasingly addressing health-related social needs. The Massachusetts Flexible Services program (Flex) is a 3-year pilot program to address food insecurity and housing insecurity by connecting Medicaid accountable care organization (ACO) enrollees to community resources.

**Objective:**

To understand barriers and facilitators of Flex implementation in 1 Medicaid ACO during the first 17 months of the program.

**Design, Setting, and Participants:**

This mixed-methods qualitative evaluation study from March 2020 to July 2021 used the Reach, Efficacy, Adoption, Implementation, Maintenance/Practical, Robust Implementation, and Sustainability Model (RE-AIM/PRISM) framework. Two Mass General Brigham (MGB) hospitals and affiliated community health centers were included in the analysis. Quantitative data included all MGB Medicaid ACO enrollees. Qualitative interviews were conducted with 15 members of ACO staff and 17 Flex enrollees.

**Main Outcomes and Measures:**

Reach was assessed by the proportion of ACO enrollees who completed annual social needs screening (eg, food insecurity and housing insecurity) and the proportion and demographics of Flex enrollees. Qualitative interviews examined other RE-AIM/PRISM constructs (eg, implementation challenges, facilitators, and perceived effectiveness).

**Results:**

Of 67 098 Medicaid ACO enrollees from March 2020 to July 2021 (mean [SD] age, 28.8 [18.7] years), 38 442 (57.3%) completed at least 1 social needs screening; 10 730 (16.0%) screened positive for food insecurity, and 7401 (11.0%) screened positive for housing insecurity. There were 658 (1.6%) adults (mean [SD] age, 46.6 [11.8] years) and 173 (0.7%) children (<21 years; mean [SD] age, 10.1 [5.5]) enrolled in Flex; of these 831 people, 613 (73.8%) were female, 444 (53.4%) were Hispanic/Latinx, and 172 (20.7%) were Black. Most Flex enrollees (584 [88.8%] adults; 143 [82.7%] children) received the intended nutrition or housing services. Implementation challenges identified by staff interviewed included administrative burden, coordination with community organizations, data-sharing and information-sharing, and COVID-19 factors (eg, reduced clinical visits). Implementation facilitators included administrative funding for enrollment staff, bidirectional communication with community partners, adaptive strategies to identify eligible patients, and raising clinician awareness of Flex. In Flex enrollee interviews, those receiving nutrition services reported increased healthy eating and food security; they also reported higher program satisfaction than Flex enrollees receiving housing services. Enrollees who received nutrition services that allowed for selecting food based on preferences reported higher satisfaction than those not able to select food.

**Conclusions and Relevance:**

This mixed-methods qualitative evaluation study found that to improve implementation, Medicaid and health system programs that address social needs may benefit from providing funding for administrative costs, developing bidirectional data-sharing platforms, and tailoring support to patient preferences.

## Key Points

**Question:**

What were the initial implementation challenges and solutions for a Medicaid accountable care organization participating in the Massachusetts Flexible Services program to address food and housing insecurity?

**Findings:**

In this mixed-methods qualitative evaluation, implementation challenges included administrative burden, COVID-19 factors that influenced screening for social needs, data tracking and sharing, and coordinating with community organizations. Adaptive solutions included administrative funding for hiring enrollment staff, bidirectional communication with community partners, new strategies to identify eligible patients, and raising clinician awareness of the Massachusetts Flexible Services program.

**Meaning:**

Future state and health systems’ programs to address health-related social needs may benefit from minimizing administrative burden, providing funding for enrollment staff and evaluation, and developing effective information-sharing platforms.

## Teaser

This qualitative study sought to understand the barriers and facilitators of Massachusetts Medicaid’s Flexible Services Program implementation in a Medicaid accountable care organization during the first 17 months from March 2020 to July 2021.

## Introduction

Health-related social needs (HRSNs) are associated with high health care utilization and costs^1,2^ and poor health outcomes.^2,3,4,5,6^ States and health care systems are increasingly recognizing the importance of addressing unmet HRSNs and implementing interventions to address these needs.^7,8^ Evaluating the effectiveness of HRSN interventions has been limited by a lack of implementation data, including details on program delivery designs and adaptations made to tailor programs to the needs of defined populations.^9,10,11^

A focus on value-based care and the proliferation of alternative payment models has further fueled the implementation of systematic interventions to address HRSNs.^12,13^ As of July 2021, 12 state Medicaid programs were using accountable care organizations (ACOs).^14^ The Centers for Medicare & Medicaid Services (CMS) encourages these ACOs to address HRSNs systematically through universal screening and referral, and partnerships with community-based organizations that specialize in social resource provision.^15,16^ Some states, including Massachusetts,^17,18^ have received Medicaid Section 1115 waivers to support health system linkages with community-based social services organizations to provide resources to members with identified HRSN.^19^ While CMS requires states to conduct and report evaluations of their waiver programs, these typically do not include evaluation of program implementation, which is critical for understanding why programs might succeed or fail and for informing future policy decisions and implementation efforts.^10,20,21,22^

In January 2020, Massachusetts Medicaid (MassHealth) launched a 3-year pilot of the Flexible Services program (Flex).^23^ The program provided $149 million statewide to ACOs to partner with social services organizations to provide nutrition and housing-related services to enrollees with food or housing insecurity and substantial health needs. Flex was not designed as an entitlement benefit or covered service for all eligible ACO enrollees; instead, it was meant to supplement existing benefit programs by providing a limited amount of additional funding to each ACO. While other ACO programs and funding supported screening and referral for social needs, the Flex program’s unique contribution was direct payment for social needs services (eg, food boxes or meals to support individuals with food insecurity). The objective of this qualitative study was to use mixed methods to conduct an implementation evaluation of the first 1.5 years of Flex (March 2020 to July 2021) in a single MassHealth ACO, guided by the Reach, Efficacy, Adoption, Implementation, Maintenance/Practical, Robust Implementation, and Sustainability Model (RE-AIM/PRISM).^24^

## Methods

This mixed-methods qualitative evaluation study was conducted in 2 large hospitals and 5 community health centers in the Mass General Brigham (MGB) Medicaid ACO in Boston, MA from March 2020 to July 2021. The mixed-methods approach used quantitative health system data to assess HRSN screening and Flex enrollment and qualitative interviews with health system staff and Flex enrollees to understand implementation challenges and successes. This evaluation was conducted as part of a longitudinal quasi-experimental study (LiveWell/ViveBien) designed to assess the association of Flex with the health and health care utilization of MGB community health center patients. The research team was not involved in the design or implementation of Flex. Study procedures were approved by the MGB institutional review board on August 27, 2019. All participants provided verbal informed consent for participation and the use of the content of their interviews (eg, quotations) in research reports and publications. This study adheres to the Standards for Reporting Qualitative Research (SRQR)^25^ reporting guideline.

### Flexible Services Program

Two categories of services are funded through Flex: nutrition support (food vouchers, food boxes, medically tailored meals) and housing support (assistance with affordable housing applications, utility bills). The ACOs were encouraged, but not required, to partner with local social service organizations (SSOs) and establish financial contracts to compensate SSOs for service delivery.^26^ MassHealth provided examples for structuring payment contracts with SSOs (eg, fee for service, prospective lump sum, bundle) but did not provide templates, requirements, or formal support for these contracts. While some ACOs provided Flex services internally (eg, hospital-based food pantries), the majority of services were delivered by SSOs.^26^ Flex enrollment was designed to occur at the individual level, but more than 1 individual in a household could be enrolled. Eligibility criteria were: (1) enrollment in a MassHealth ACO, (2) food or housing insecurity identified by screening or clinical encounter, and (3) complex physical or behavioral health need (eg, obesity, uncontrolled diabetes, uncontrolled depression), high emergency department use (ie, at least 2 visits in 6 months or at least 4 visits in 1 year), or high-risk pregnancy. During the period of this evaluation, MGB planned for approximately 1900 enrollments into Flex SSOs that served the hospitals and community health centers in this study. A detailed description of Flex is included in the eMethods in Supplement 1.

In anticipation of the 2020 start of Flex, Medicaid ACOs were required to begin annual HRSN screening in 2018. The MGB system began systematic screening in March 2018 using electronic tablet–delivered surveys in primary care practices. Screening was prompted in the electronic health record (EHR) for all ACO members at the time of a clinical visit every 12 months. Screeners were either self-administered by patients or administered by health center staff. Screening data were recorded in a specific, structured EHR module. Food insecurity was assessed with the validated 2-item US Department of Agriculture (USDA) screener.^27^ Housing insecurity was assessed using 3 items developed from prior literature.^28,29^

### Implementation Measures

The RE-AIM/PRISM framework was used to evaluate Flex implementation from the perspective of the Medicaid ACO.^24^ Table 1 summarizes the application of the framework in the current study, including definitions, data sources, and measures for RE-AIM/PRISM constructs. Quantitative data was used to measure reach and qualitative data to assess implementation, adoption, perceptions of effectiveness, and the PRISM constructs of internal and external context.

**Table 1.** Application of RE-AIM/PRISM for a Mixed-Methods Implementation Evaluation of the MassHealth Flexible Services Program in the Mass General Brigham Accountable Care Organization

RE-AIM/PRISM outcomes	Glasgow et al,^24^ 2019 definition	Data source	Study Measure (March 2020-July 2021)
Reach	The “number, proportion, and representativeness of individuals” who participated in the program.	EHR	Number and proportion of Medicaid ACO patients^a^ screened for food and housing insecurity (March 2020-July 2021) Number and proportion of patients enrolled in Flex (March 2020-July 2021) Representativeness of Flex enrollees vs overall ACO population Number and proportion of Flex enrollees who received services
Effectiveness	“The impact of [the program] on important outcomes.”	Patient interviews, staff interviews	Changes in patients’ food and housing security Changes in patients’ dietary intake and health behaviors Changes in patients’ level of stress
Adoption	“Reasons for adoption or non-adoption” of program components.	Staff interviews	Barriers and facilitators of social needs screening Barriers and facilitators of Flex referrals Barriers and facilitators of Flex enrollment
Implementation	“Fidelity to the various elements of [the program's] protocol, including consistency of delivery as intended” and “adaptations made.”	Patient interviews, staff interviews, EHR	Fidelity to screening, enrollment, and program delivery as planned Barriers/facilitators of implementation Adaptations made to improve implementation
Maintenance	“The extent to which…a program or policy becomes institutionalized.”	Staff interviews, EHR	To be assessed in future implementation evaluation
Internal context	Internal environment (eg, organizational structure, characteristics, capacity; patient characteristics)	Patient interviews, hospital staff interviews	Health-system factors that inhibit or facilitate implementation
External context	External environment (eg, policies, guidelines, external events)	MGB staff interviews	MassHealth policies Public health context (eg, COVID-19 pandemic)

Abbreviations: ACO, accountable care organization; EHR, electronic health record; MassHealth, Massachusetts Medicaid; MGB, Mass General Brigham; RE-AIM/PRISM, Reach, Effectiveness, Adoption, Implementation, Maintenance/Practical, Robust Implementation, and Sustainability Model.

^a^ Patients were aligned to 2 large hospitals in the MGB Medicaid ACO and their affiliated community health centers.

Reach of Flex was assessed using 3 metrics. First, completion of the annual health system-based screener for food and housing insecurity (numerator) using EHR data was examined among adult and pediatric Medicaid ACO patients (denominator) from March 2020 to July 2021. The second reach metric was the number and proportion of Medicaid ACO patients enrolled in Flex during this time and the demographic characteristics (age, sex, and self-reported race and ethnicity) of Flex enrollees compared with the overall ACO population. The third metric was the health conditions of Flex enrollees documented by staff in an EHR Flex enrollment form as eligibility criteria.

Implementation, adoption, perceptions of effectiveness, and internal and external contextual factors based on RE-AIM/PRISM were assessed using qualitative interviews with key implementation personnel in the health system and adult Flex enrollees. Given the early stage of the Flex pilot, maintenance was not assessed. Staff was invited to participate in interviews using purposive sampling based on their Flex responsibilities; this included ACO staff involved in the design and roll-out of Flex (eg, individuals employed centrally by the ACO), as well as hospital and community health center staff who implemented Flex (eg, Flex program managers and enrollment staff). Staff was invited for interviews by a PhD-level investigator (J.L.M.), who explained her role as a researcher and had no formal working relationship with interviewees. A semistructured qualitative interview guide elicited the staff’s role in Flex and descriptions of implementation barriers and facilitators, including internal and external contextual factors (eFigure in Supplement 1). Staff did not receive remuneration. To protect participants, job roles were described using broad definitions (ie, ACO staff, hospital staff), and interview results that could potentially identify individuals, including specific quotations, were excluded.

Flex enrollees who participated in qualitative interviews were LiveWell/ViveBien study participants who enrolled in Flex before July 2021. Participants were invited using purposive sampling based on sex, language (English, Spanish), health center affiliation, and receipt of food resources vs housing resources. Interviews were conducted in English or Spanish. The semistructured interview guide for Flex enrollees targeted aspects of program implementation (eg, barriers, facilitators, satisfaction) and perceived effectiveness (eg, changes in food or housing insecurity or health behaviors) (eFigure in the Supplement). Enrollees received $25 for participation.

### Quantitative Data and Statistical Analyses

Screening for food and housing insecurity was recorded in the EHR. Any screening completed between March 2018 and July 2021 was included. To determine Flex enrollment outcomes (eg, whether a patient received services, declined, or was unreachable), a combination of data sources was used, including the following: (1) formal enrollment forms in the EHR, (2) enrollment and service provision data entered in an electronic data sharing platform that the ACO adapted for bidirectional ACO-SSO communication, and (3) quarterly enrollment reports maintained by ACO project management staff, which included information from SSO-specific Flex tracking and reporting documents. Demographic characteristics (age, sex, and self-reported race and ethnicity) for ACO and Flex enrollees were obtained from the EHR and summarized using Stata statistical software, version 16.0 (StataCorp).

### Qualitative Interviews and Thematic Analysis

All qualitative interviews were conducted via phone or video platform, audio-recorded, and transcribed. Qualitative data were coded and analyzed using Dedoose software, version 9.0.54 (SocioCultural Research Consultants, LLC), and the framework method thematic analysis approach.^30^ The research team identified themes based on content in the interview guides (eFigure in Supplement 1). Two trained bilingual research assistants independently coded all transcripts. Discrepancies were resolved as needed with input from a PhD-level researcher (J.L.M.). Saturation was determined when novel themes no longer emerged. Themes and results were reviewed with the research team and select interviewees to ensure accuracy and face validity.

## Results

### Annual Screening for Food and Housing Insecurity

Of the 67 098 Medicaid ACO patients in the sample from March 2020 to July 2021, 38 442 (57.3%) completed at least 1 screening for food insecurity and housing insecurity between 2018 and 2021; 10 730 (16.0% of all ACO enrollees) screened positive for food insecurity; and 7401 (11.0% of all ACO enrollees) screened positive for housing insecurity at least once (Table 2). These rates were similar for adults and children. The rates of screening and food and housing insecurity were higher among Flex enrollees: 573 (87.1%) adults and 158 (91.3%) children completed at least 1 screener; 61.6% of adults and 49.1% of children screened positive for food insecurity, and 35.6% of adults and 26.0% of children screened positive for housing insecurity.

**Table 2.** Completion of Annual Screening for Food and Housing Insecurity by Adult and Pediatric Medicaid Accountable Care Organization Enrollees

Characteristic	Medicaid ACO enrollees^a^ (March 2018-July 2021)				
	Total	Adults (≥21 y)	Adults enrolled in Flex^b^	Children (<21 y)	Children enrolled in Flex^b^
No.	67 098	40 616	658	26 482	173
At least 1 SDOH screener complete, No. (%)^c^	38 442 (57.3)	21 575 (53.1)	573 (87.1)	16 867 (63.7)	158 (91.3)
Ever screened positive for food or housing insecurity, No. (%)	14 059 (21.0)	8674 (21.4)	449 (68.2)	5385 (20.3)	95 (54.9)
Ever screened positive for food insecurity, No. (%)	10 730 (16.0)	6762 (16.6)	405 (61.6)	3968 (15.0)	85 (49.1)
Ever screened positive for housing insecurity, No. (%)	7401 (11.0)	4734 (11.7)	234 (35.6)	2667 (10.1)	45 (26.0)

Abbreviations: ACO, accountable care organization; Flex, Flexible Services program; SDOH, social determinants of health.

^a^ Medicaid ACO enrollees were patients enrolled in the Mass General Brigham (MGB) ACO who were aligned to 2 of the largest hospitals in the health system and their affiliated community health centers.

^b^ Eligibility criteria for Flex included (1) enrollment in MGB Medicaid ACO; (2) food or housing insecurity identified by screening or clinical encounter; and (3) a complex health condition (eg, uncontrolled diabetes, depression).

^c^ Screening was completed during primary care health visits or through contact with patients’ primary care clinicians from March 2018 (beginning of annual social needs screening for Medicaid ACO patients) through July 2021 (end of the current analysis). These results reflect screening that was documented in a designated social needs screening module of the electronic health record (EHR). Assessment of social needs may have also occurred via informal conversations between patients and clinicians or other health center staff and documented in the EHR in nonsystematic ways; those assessments are not reflected here.

### Enrollment and Receipt of Flexible Services

A total of 658 (1.6%) adult patients (mean [SD] age, 46.6 [11.8] years) and 173 (0.7%) pediatric patients (<21 years; mean [SD] age, 10.1 [5.5]) were enrolled in Flex between March 2020 and July 2021 (Table 3). Of these 831 people, 613 (73.8%) were female participants, 444 (53.4%) were Hispanic/Latinx participants, and 172 (20.7%) were Black participants. Obesity was the condition most frequently documented by staff to fulfill Flex enrollment criteria (428 [65.1%] adults, 103 [59.5%] children) followed by uncontrolled depression (142 [21.6%] adults) and developmental disorders (31 [17.9%] children) (eTable in Supplement 1).

**Table 3.** Demographic Characteristics of Medicaid Accountable Care Organization Enrollees and Flexible Services Program Enrollees

Characteristic	Medicaid ACO enrollees^a^ (March 2020-July 2021)				
	Total	Adults (≥21 y)	Adults enrolled in Flex^b^	Children (<21 y)	Children enrolled in Flex^b^
No.	67 098	40 616	658	26 482	173
Age, mean (SD)	28.8 (18.7)	41.2 (13.0)	46.6 (11.8)	9.9 (5.9)	10.1 (5.5)
Sex, No. (%)					
Female	38 809 (57.8)	25 696 (63.3)	539 (81.9)	13 113 (49.5)	74 (42.8)
Male	28 289 (42.2)	14 920 (36.7)	119 (18.1)	13 369 (50.5)	99 (57.2)
Ethnicity					
Hispanic	24 461 (36.5)	12 503 (30.8)	360 (54.7)	11 958 (45.2)	84 (48.6)
Non-Hispanic	34 021 (50.7)	24 334 (59.9)	284 (43.2)	9687 (36.6)	69 (39.9)
Unknown	8616 (12.8)	3779 (9.3)	14 (2.1)	4837 (18.3)	20 (11.6)
Race^c^					
American Indian or Alaska Native	148 (0.2)	107 (0.3)	2 (0.3)	41 (0.2)	0 (0)
Asian	2925 (4.4)	1983 (4.8)	6 (0.9)	942 (3.6)	4 (2.3)
Black	10 608 (15.8)	7424 (18.3)	143 (21.7)	3180 (12.0)	29 (16.8)
Native Hawaiian or Pacific Islander	57 (0.1)	39 (0.1)	0 (0)	18 (0.1)	0 (0)
White	25 077 (37.4)	17 983 (44.3)	169 (25.7)	7094 (26.8)	44 (25.4)
Other^d^	17 247 (25.7)	8444 (20.8)	210 (31.9)	8803 (33.2)	70 (40.5)
Multiracial	1564 (2.3)	803 (2)	8 (1.2)	761 (2.87)	6 (3.5)
Unknown	9476 (14.1)	3833 (9.4)	120 (18.2)	5643 (21.3)	20 (11.6)

Abbreviations: ACO, accountable care organization; Flex, Flexible Services program.

^a^ Patients enrolled in the Mass General Brigham (MGB) ACO who were aligned to 2 of the largest hospitals in the health system and their affiliated community health centers.

^b^ Eligibility criteria for Flex enrollment included (1) enrollment in MGB Medicaid ACO; (2) food or housing insecurity identified by screening or clinical encounter; and (3) a complex health condition (eg, uncontrolled diabetes, depression).

^c^ Race was self-reported in the electronic health record data. All categories from the electronic health record are included in the table.

^d^ Enrollees self-identified as “other” race; 82% of enrollees in this category reported Hispanic ethnicity.

Of 658 adults enrolled in Flex, 584 (88.8%) received services, 14 (2.1%) declined services after enrollment, and 60 (9.1%) were unreachable or had no documented service outcome. Of those who received services, 505 (76.7%) received nutrition support, 87 (13.2%) received housing support, and 21 (3.2%) received both. Of 173 pediatric Flex enrollees, 143 (82.7%) received services, 6 (3.5%) declined services after enrollment, and 24 (13.9%) were unreachable or had no documented service outcome. Of those who received services, 114 (65.9%) received nutrition support, 35 (20.2%) received housing support, and 6 (3.5%) received both.

The Figure displays the number of new Flex enrollees each month between March 2020 and July 2021. Events that occurred within the health system (eg, staffing changes in the ACO) and external to the health system (eg, COVID-19 pandemic, MassHealth policies) are superimposed on the Figure to demonstrate how they may have influenced enrollment. Flex roll-out in March 2020 coincided with the state-mandated COVID-19 lockdown; this external contextual factor contributed to lower enrollment from March 2020 to May 2020. External program adaptations included MassHealth’s enactment of 2 policy modifications. First, in early 2020, MassHealth removed the in-person screening requirement to enroll patients into Flex. Second, in June 2020, MassHealth allowed ACOs to use Flex funding for administrative costs, such as salaries for enrollment staff. Internal program adaptations that coincided with increased enrollment included: execution of additional SSO contracts, development of hospital-specific strategies to identify eligible patients and improve administrative workflow, an ACO-led campaign to increase clinicians’ awareness of Flex and assist with local problem-solving for screening and enrollment, and hiring additional enrollment staff (Figure).

**Figure.**
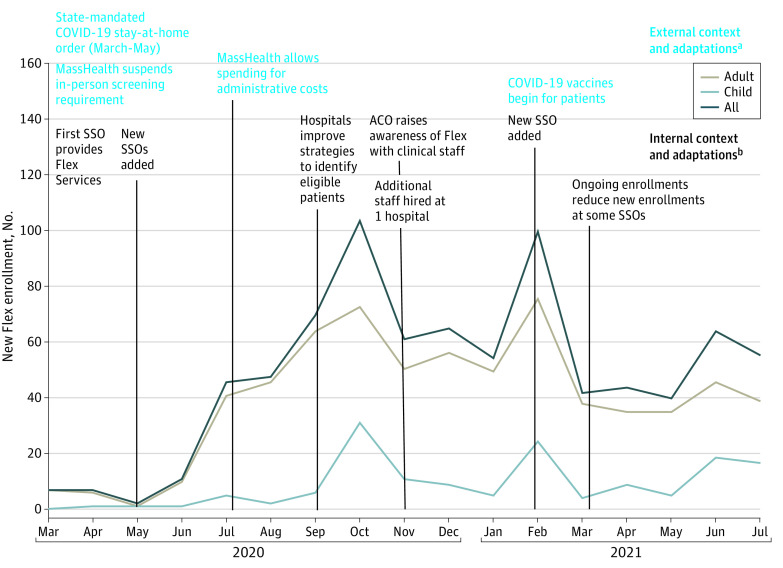
Monthly Flexible Services Program Enrollments of Medicaid Accountable Care Organization (ACO) Patients Aligned to 2 Large Hospitals This mixed-methods qualitative study occurred in a Massachusetts ACO from March 2020 to July 2021 during the first 17 months of program implementation. The labels on this line graph show the factors that may have influenced the pace of enrollments. Abbreviations: Flex, Flexible Services program; MassHealth, Massachusetts Medicaid; SSO, social service organization. ^a^External environmental factors (eg, policies, guidelines, external events) and implementation adaptations that influenced new enrollments into Flex. ^b^Internal environmental factors (eg, organizational structure, characteristics, capacity) and implementation adaptations that influenced new enrollments into Flex.

### Implementation Barriers, Facilitators, and Adaptive Solutions

Table 4 presents Flex implementation challenges and adaptive solutions as reported by health system staff (n = 15) and Flex enrollees (n = 17). Staff reported challenges in the administration of Flex that included complex eligibility requirements, time-intensive enrollment paperwork, and burden associated with identifying appropriate patients (eg, screening, EHR review) and providing follow-up support (eg, communicating with SSOs, tracking outcomes). In addition to screening positive for food or housing insecurity, ACO enrollees had to meet health criteria to be eligible for Flex; these criteria included both having certain health diagnoses and needing improvement or control within that condition (eg, having uncontrolled type 2 diabetes vs simply having type 2 diabetes). The complexity of verifying these eligibility criteria created a substantial burden for enrollment staff. The 2 hospital entities differed in their abilities to accommodate the Flex administrative burden, highlighting the importance of preexisting staff availability as an internal contextual factor. One hospital entity redeployed existing social work and case management staff for Flex enrollment early in 2020, but the other lacked staffing flexibility and benefited from the MassHealth policy change allowing Flex funding to cover administrative costs. The ACO staff described developing new methods for automating and systematizing Flex-related workflows as the program was rolled out. Staff reported that adaptations to reduce the administrative burden took approximately 1 year.

**Table 4.** Staff-Reported and Patient-Reported Barriers, Facilitators, and Adaptive Solutions in the First 17 Months^a^ of Implementation of the Flexible Services Program in the Mass General Brigham Accountable Care Organization

Implementation components	Barriers and challenges	Facilitators and adaptive solutions
Flex administration	Documentation burden: “We had a really big barrier of getting people to embrace the program…there’s a lot of documentation,” said an ACO staff member.^b^ Hospital differences in pre-existing capacity: “One of the groups said, ‘We’re just going to leverage our social workers.’ And they found a lot of success doing that. Other groups don’t have as many social workers, so they can’t implement the process that way,” said an ACO staff member. “Some hospitals and clinics will have much more resources like they will have analytic support, say, while some of the smaller organizations don’t. And that also goes into the whole administrative thing—who can access analytic support. Not only just actual bodies but things such as identifying patients. Some [sites] need access to more central resources,” said an ACO staff member.	Automating workflows: “The team developed a report where all of that chart review populates itself. So all the scores, the BMI value, etc., would automatically be pulled from the patient’s chart. So we were able to cut down how much chart review we were doing. And it’s not as overwhelming,” said a hospital staff member.^c^ MassHealth adding funding for administrative purposes: “We’ve been able to hire 3 positions using administrative funding. If that had been built into the program up-front, we probably could have had the appropriate staff members in place to do the work,” said a hospital staff member.
Flex enrollment procedures	Complex eligibility requirements: “When we first started doing this work, we were leaning on care management teams to help us to identify folks that would be a good fit…that was our primary strategy. I think the challenge there is that when we think about across the system, we know that we could also use different strategies such as running reports…perhaps that would help us have a broader capture,” said a hospital staff member. “Eligibility for the program depends on the patient's sociomedical condition and their status as a patient of [hospital name], and in order to qualify, they must have a significant health risk that can be improved by having access to food or has been identified as food insecure,” said a hospital staff member.	Local enrollment strategies and priorities: “One hospital is like, ‘Let’s try to get everyone in,’ and really focused on food insecurity, and the other hospital is saying, ‘We want to enroll our highest risk patients, who can really, really benefit from this program’…They’re really focusing on the family unit,” said an ACO staff member.
Health system partnerships with SSOs	Cross-sector differences: “Working with an outside organization is hard. Yes, we pay them. And yes, they're accountable to this program. But they're not at our desk with us. They're not in health care, which is very, very different than any other organization out there. So there's been a lot of work around setting expectations with the social service organizations,” said an ACO staff member. Challenges with contracts and payment structure: “We're not paying [SSOs] unless they're—all of our payments are based on members. It's not like we give up-front dollars to them. So if we say we're going to refer 100 members, and we only refer 50, they're only getting about 50% of the money that we said that we were going to pay them. And that's not fair, right? These are nonprofits. And they're doing a lot of hard work. And we want to pay them the full amount,” said an ACO staff member. Communication between health system and SSOs: “We get a lot of concerns from our providers who refer people and then hear back from patients that they haven't heard from the (SSOs), or that the process with the (SSOs) is disjointed,” said a hospital staff member. Inconsistent or redundant documentation: “A lot of these (SSOs) have their own systems and their own way of tracking, and we’re essentially making them use ours that is counterproductive or counterintuitive to their own system,” said a member of hospital staff.	Structured communication/contact: “We have meetings with [SSO name] once a month. And we do a secure email chain. We set that up once a week and just kind of go over, in real time, any issues. And then we have meetings where we talk about any new goals, any new updates. So I think communication with them has gone really well,” said a hospital staff member. Local selection or choice in partnerships: “For each [hospital name] site, we worked with them to pick the SSOs in their area that they felt were best for their population,” said an ACO staff member. Bidirectional data-sharing platform: “Maybe because I'm a true optimist, but it was just like, ‘OK [data sharing platform company name], by the time you're done with us, you could work with literally any SSO and any hospital organization.’ So it's great that we're ironing out the kinks now. But I think this was just a thing that no one thought of in the beginning,” said an ACO staff member. SSO communication with hospital case managers: “I know that with [SSO name], they keep in touch with the patients. For example, for the inactive patients, the coordinator, he will call those patients to see why they are inactive. And if he's unable to reach the patients, he passes them on to [hospital case manager name] and we reach out to those patients to see why they are inactive and what support we can offer,” said a hospital staff member.
COVID-19 pandemic	Disruptions to routine care and SDOH screening: “The SDOH screening, which was originally done using an iPad in the practice when a patient would come in for a visit—that went away. Because of COVID-19, the practices were partially open. Patients weren't coming in…So when COVID-19 hit, we had to change our expectations. And to this very day, we are struggling to screen as many patients as we used to on social determinants of health. And if we don't screen, we can't identify needs. And if we don't identify a need, we can't enroll,” said a hospital staff member. “COVID disrupted the way of doing everything,” said a hospital staff member.	Responsive changes in program requirements: “So MassHealth, they waived the requirement for in-person screening of patients to enroll them. We can do the screening by calling the patient over the phone…If they did not waive that requirement, I don't think we would have enrolled anyone last year,” said a hospital staff member.
Patient experiences with nutrition services	Food not aligned with patient needs or preferences: “It's not food they were used to. I tried to let them know what types of food they would be receiving when I was enrolling them, so squash soup, chicken, vegetables, tofu. A patient called that she didn't like tofu. So things like that, that they wanted to give it a try, but they just didn't like it. So they would either throw it out or give it to other family members,” said a hospital staff member. Barriers to accessing services: “The problem is that I go in [to work] at 9, and the food truck arrives from 11 to 12. It is very difficult at that time. I mean, I have to get away from work, and during the break, I don’t have time, I am a bit far away,” said a patient receiving a food truck voucher. Suboptimal quality of services: “Sometimes they [produce items] were a day or 2 away from expiring…many of the things would go bad the next day if you did not use them,” said a patient receiving a produce box.	Patients’ ability to choose food: “That you can pick the stuff you want, like your own fruits and vegetables, because they have other programs that you can go and pick up fruits and vegetables, but they have it already packed up. And sometimes people don't eat certain stuff. So what I like about this is I could pick what I want to eat and what I need,” said a patient receiving a food voucher. Exposure to healthy food: “I've eaten Brussels sprouts, and I've eaten things that I haven’t eaten before, and I'm starting to get a different palate, which is a good thing,” said a patient receiving medically tailored meals. Increased motivation for self-management: “I feel like it's kind of like a push on my back. Like, ‘You've got this. You have this opportunity. You just had your GI appointment, and some of these changes are going to be beneficial…or at least try to find out.’ And then it's like, ‘You know what? I do have [Flex]. Let's do it,’” said a patient receiving a food voucher.
Patient experiences with housing services	Lack of affordable housing options for unhoused patients: “She [housing SSO worker] was telling me about where I was at on the [public housing] list, that they are doing 2007 now, or something. So if they're doing 2007 now, I'll be another 20 years (before receiving housing),” said a patient receiving housing application support. Difficulty maintaining contact with SSO staff: “She [housing SSO worker] tried to get me to meet her somewhere, but I forgot the day to go meet her. And then I don’t really have a phone number. So getting back in touch with her is a problem,” said a patient receiving housing application support.	Coordination between SSOs and community organizations: “They helped me out with the gas bill…someone from [housing SSO] went to [another community organization] and they applied for me…and it’s a program that helps you with gas and other resources for parents,” said a patient receiving support with the gas heating bill.

Abbreviations: ACO, accountable care organization; BMI, body mass index; Flex, Flexible Services program; GI, gastrointestinal; MassHealth, Massachusetts Medicaid; SSO, social service organization.

^a^ The first 17 months included March 2020 to July 2021.

^b^ ACO staff included staff employed centrally by the ACO (eg, Flex managers).

^c^ Hospital staff included staff employed at individual hospitals and community health centers (eg, hospital-specific Flex managers and enrollment staff).

Partnering and information-sharing with SSOs were additional complex challenges. Staff described challenges with SSOs’ financial contracts and payment models, cross-sector differences in communication style and expectations for services, and problems accessing follow-up information for referred patients. While hospitals were responsible for establishing payment contracts with SSOs, ACO staff reported difficulty estimating the volume of referrals that an SSO would receive and noted that some SSOs struggled to provide services due to pandemic-related staffing and supply challenges. Further, staff described that SSO payment contracts often involved fee-for-service invoicing which hospitals completed weeks or months after service delivery, contributing to operational and budgetary strain for SSOs.

Staff emphasized the need for improved bidirectional information sharing to communicate with SSOs and track outcomes. While a pre-existing resource platform was adapted in the first year to serve as an ACO-SSO communication and tracking platform, use of this platform added to the documentation burden for both staff and SSOs. Flex services provided internally (eg, hospital-based food pantry) were not documented in the platform. Staff described other limitations of the platform, such as overwritten historical data and cumbersome user experience. The limitations and documentation burden of the data-sharing platform were additional internal contextual factors that contributed to information-sharing challenges.

Staff noted that the COVID-19 pandemic was an external contextual factor that contributed to slow initial Flex enrollment in part because HRSN screening required in-person office visits, which declined significantly in March 2020. State and local program adaptations were critical to respond to this challenge; MassHealth discontinued the in-person screening requirement, and hospitals pushed questionnaires to patients through the EHR while also initiating new systems to identify eligible patients (eg, reports based on health eligibility criteria).

Interviews of Flex enrollees highlighted barriers to program effectiveness as well as early signals of positive health outcomes. In general, Flex enrollees reported that Flex enrollment and connecting with SSOs was feasible. Enrollees reported barriers to using nutrition services that included poor access (eg, lack of transportation), poor fit with dietary preferences, and suboptimal quality of food (eg, produce near expiration). Conversely, enrollees reported that nutrition supports improved or resolved their food insecurity, helped them make healthy dietary changes, and improved their motivation or capacity for self-management of health conditions. Enrollees who were older or had more complex health conditions expressed satisfaction with medically tailored meals or preset food boxes, whereas younger patients and patients with dependent children preferred to choose their own food. Flex enrollees with dependent children often reported that the nutrition support improved their children’s dietary intake and health as well as their own.

Enrollees receiving housing support reported overall lower satisfaction. Housing support received by interview participants included assistance with affordable housing applications and short-term assistance with utility bills. Enrollees described that, while helpful, these supports did not resolve their housing insecurity, and they had little or no change in their health or health behaviors as a result of housing support received.

## Discussion

This mixed-methods qualitative evaluation study evaluated the MassHealth Flexible Services program in 1 Medicaid ACO and identified factors that influenced its reach, adoption, implementation, and perceived effectiveness. While external contextual factors, including the COVID-19 pandemic, slowed the initial reach and implementation, program modifications during the first year improved the reach and implementation of Flex. This study characterized the who, what, and why of Flex modifications enacted by MassHealth and the ACO, which are critical implementation data that may inform future HRSN programs.^31^

Flex’s reach within the first 17 months was modest, largely due to the COVID-19 pandemic and initial administrative barriers. The number of Flex enrollments was slightly less than half of the ACO’s projection for the study period. However, the program enrolled a diverse and representative sample of the ACO population, including a high proportion of Hispanic/Latinx and Black patients. Approximately 1 in 5 Flex enrollees were children, highlighting that programs targeting individuals with HRSN and health needs benefit children as well as adults. Although Flex was designed to serve individuals, the qualitative findings suggest there were also benefits at the household level. More than 80% of enrolled patients received the intended food or housing support services. In comparison, a study of an intervention requiring patients with HRSN to contact a community resource helpline on their own (vs being referred through a health system pipeline) demonstrated only 36% received services.^32^ With obesity as the most prevalent qualifying health condition, Flex has the opportunity to influence critical risk factors in people with high cancer and cardiometabolic disease risk.^33,34^ In qualitative interviews, Flex enrollment was reported to be feasible, and those receiving nutrition services largely reported increased food security—a potential early signal of program effectiveness.

The study findings underscore the challenges of implementing systematic HRSN screening. In this well-resourced Medicaid ACO, only 57% of ACO enrollees completed a screening between 2018 and 2021. Even among Flex enrollees, screening rates were not 100%, indicating that HRSN were also assessed in informal ways (eg, conversations with clinicians or enrollment staff). While systematic screening efforts are required by an increasing number of state Medicaid agencies,^35^ challenges are well documented, including resource constraints^36,37^ and time burden for patients and staff.^37,38^

Two persistent challenges for Flex implementation—administrative burden and limited data-sharing infrastructure—yield important recommendations for future HRSN interventions, including the Flex program, which was extended through December 2027 as part of Massachusetts’ recent 1115 waiver renewal. To address the administrative burden of cross-sector initiatives, Medicaid agencies could offer administrative funding to health systems and community partners at the start of enrollment, as well as structured support for cross-sector partnerships, such as providing contract templates and best practice recommendations. Increased investment in technological assistance and infrastructure to support data sharing and outcomes reporting is critical. A recent national survey of state Medicaid agencies found that few states with active Medicaid HRSN screening or intervention protocols had systems to link data between Medicaid, health systems, and community partners.^36^ Data sharing between clinical and community partners and increased administrative burden were also substantial implementation challenges in the 2017-2022 Accountable Health Communities initiative to address social needs of Medicare recipients.^39^ As has been the case with health information exchange,^40^ coordination across competing platforms and incompatible systems present challenges that are compounded by the need for connections between the highly regulated health sector and the less-regulated social services sector. Investment in standardized data management technologies, designed with the needs of community and health system users in mind, could decrease these challenges and facilitate more rigorous evaluation of HRSN interventions.

### Limitations

First, the evaluation focused on 1 ACO in a well-resourced urban health system. Results may not be representative of all Massachusetts’ ACOs, and findings should be considered alongside others’ Flex experiences. While a statewide evaluation of Massachusetts’s 1115 waiver programs is planned,^41^ the current study provides an early assessment of the associations between HRSN screening, Flex program enrollment, and patient experiences that can inform ongoing efforts to implement and sustain Flex and similar programs. Second, this study included perspectives of stakeholders inside the ACO (ie, staff, patients), but not outside entities (eg, MassHealth, SSOs). Finally, the COVID-19 pandemic and public health emergency measures during the study period influenced HRSN in myriad ways; while illness and reduced wages may have increased HRSN for some individuals, cash payments and evictions moratoriums may have reduced HRSN for others. Therefore, early Flex experiences may not be representative of program implementation when no public health emergency is in place.

## Conclusions

The early implementation challenges of MassHealth’s Flexible Services program identified in this mixed-methods qualitative evaluation study, including program administration, cross-sector partnerships, and information sharing and data sharing, are similar to other Medicaid HRSN programs.^21,36,42^ For optimal reach, adoption, and implementation in diverse low-income populations, states, and health systems should provide funding to address administrative burdens, design effective data-sharing platforms, tailor support programs to patient preferences, and assess implementation outcomes during program delivery.

## Notes

Supplement 1. **eMethods.** The MassHealth Flexible Services Program **eTable.** Health conditions documented by health center staff as eligibility criteria for enrollment into the Flexible Services program for patients aligned to 2 large hospitals in the Mass General Brigham Accountable Care Organization from March 2020 to July 2021. **eFigure.** Semi-structured interview scripts for qualitative interviews with health system staff and Flexible Services program enrollees

Supplement 2.
Data Sharing Statement
