# Supplementary material for: Assessment of the Massachusetts Flexible Services Program to Address Food and Housing Insecurity in a Medicaid Accountable Care Organization
Source: JAMA Health Forum. 2023 Jun 2;4(6):e231191. doi: 10.1001/jamahealthforum.2023.1191 (PMC10238945; doi:10.1001/jamahealthforum.2023.1191)
Supplement: Supplement 1. — eMethods. The MassHealth Flexible Services Program eTable. Health conditions documented by health center staff as eligibility criteria for enrollment into the Flexible Services program for patients aligned to 2 large hospitals in the Mass General Brigham Accountable Care Organization from March 2020 to July 2021. eFigure. Semi-structured interview scripts for qualitative interviews with health system staff and Flexible Services program enrollees [file jamahealthforum-e231191-s001.pdf]

Supplemental Online Content

McCurley JL, Fung V, Levy DE, et al. Assessment of the Massachusetts Flexible Services program to address food and housing insecurity in a Medicaid accountable care organization. *JAMA Health Forum.* 2023;4(6):e231191 doi:10.1001/jamahealthforum.2023.1191

eMethods. The MassHealth Flexible Services Program

eTable. Health conditions documented by health center staff as eligibility criteria for enrollment into the Flexible Services program for patients aligned to 2 large hospitals in the Mass General Brigham Accountable Care Organization from March 2020 to July 2021.

eFigure. Semi-structured interview scripts for qualitative interviews with health system staff and Flexible Services program enrollees

This supplemental material has been provided by the authors to give readers additional information about their work.

© 2023 McCurley JL et al. *JAMA Health Forum.*

eMethods: The MassHealth Flexible Services Program

As part of the 5-year, $1.8 billion Massachusetts Delivery System Reform Incentive Payment Program (DSRIP) authorized through a 1115 waiver in 2018, Massachusetts Medicaid (MassHealth) launched a 3-year pilot of the Flexible Services Program ("Flex") starting in 2020.[1] This program provided $149 million statewide to Accountable Care Organizations (ACOs) to partner with community-based social services organizations (SSOs) to provide nutrition and housing-related services to enrollees with food or housing insecurity and significant health needs. Flex was not designed as an entitlement benefit or covered service to be provided to all eligible ACO members. Instead, it was meant to supplement existing benefit programs by providing a limited amount of additional funding to each ACO. While many ACOs had preexisting programs and funding to support screening and referral for social needs, the Flex program's unique contribution was the direct payment for social needs services. ACOs were encouraged, but not required, to partner with SSOs and establish financial contracts to pay for the social services provided to Flex enrollees.

This eMethods supplement summarizes the eligibility criteria, allowable uses of funding, reporting requirements, and other guidance to ACOs from MassHealth, as well as additional program details specific to the ACO that was the focus of this publication (Mass General Brigham, Boston, MA).

Eligibility for Flexible Services.
To be eligible to receive Flexible Services, ACO members were required be experiencing at least one of three social risk factors: (1) experiencing homelessness; (2) at risk of experiencing homelessness, or (3) at risk for nutritional deficiency or imbalance due to food insecurity, as determined by either screening measures or a clinical encounter. In addition, members had to meet at least one of five health needs-based criteria. MassHealth provided the following criteria to define each of these conditions:

Social Risk Factors
1. Experiencing homelessness:
   A. An individual who lacks a fixed, regular, and adequate nighttime residence,
   B. An individual who will imminently lose their primary nighttime residence, or
   C. Any individual who is fleeing domestic violence or other dangerous or life-threatening conditions and has no other residence.

2. At risk of homelessness:
To qualify as eligible under this risk factor, a member must be experiencing Part A *and* at least one factor of Part B.
   A. An individual who does not have sufficient resources or support networks immediately available to prevent them from moving to an emergency shelter or another place not meant for human habitation or a safe haven; *and*
   B. Meets one of the following conditions:
      1. Has moved because of economic reasons 2 or more times during the past 60 days;
      2. Is living in the home of another person because of economic hardship;
      3. Lives in a hotel or motel not paid for by other social services;
      4. Lives in a single-room occupancy or efficiency apartment unit in which there reside more than two persons;
      5. Has a past history of receiving services in a publicly funded institution;
      6. Otherwise lives in housing that has characteristics associated with instability and an increased risk of homelessness.

© 2023 McCurley JL et al. *JAMA Health Forum.*

3. At risk for nutritional deficiency or nutritional imbalance due to food insecurity:
Food insecurity is defined as:

A. Having limited or uncertain availability of nutritionally adequate, medically appropriate, and/or safe foods; or

B. Limited or uncertain ability to acquire or prepare acceptable foods in socially acceptable ways.

Health Needs-Based Criteria

To receive Flexible Services, an individual must meet at least one of five health needs-based criteria below, designated by MassHealth. ACOs could choose to focus their Flex population on, or within, one of the five criteria.

1. A behavioral health need (mental health or substance use disorder) requiring improvement, stabilization, or prevention of deterioration of functioning;
2. A complex physical health need, defined as a persistent, disabling, or progressively life-threatening physical health condition (e.g., diabetes, hypertension) requiring improvement, stabilization, or prevention of deterioration of functioning;
3. A need for assistance with one or more Activities of Daily Living or Instrumental Activities of Daily Living;
4. Repeated incidents of emergency department use (defined as 2 or more visits within six months, or 4 or more visits within a year); or
5. The individual is pregnant and experiencing high risk pregnancy or complications associated with pregnancy.

The presence of a diagnosis or condition alone was not sufficient to satisfy the requirements of health needs-based criteria 1-3. A need that was related to the diagnosis or condition was also required. For example, a member with a diagnosis of Generalized Anxiety Disorder was not automatically eligible based on the presence of the condition; the ACO was required to demonstrate a need for improvement, stabilization, or prevention of deterioration of the condition. For example, demonstrating this member's need for medication or psychotherapy sessions to manage their Generalized Anxiety Disorder would fulfill the needs-based criteria for a diagnosis.

In the Mass General Brigham (MGB) ACO participation plan for Flex, the following priority areas for health needs-based criteria were included:

1. Behavioral health conditions
    a. E.g., uncontrolled anxiety, uncontrolled depression, substance use disorders
2. Complex physical health conditions, including:
    - Obesity (for adults, BMI >30 kg/m^2; for children, BMI >95th percentile)
    - Hypertension, uncontrolled
    - Diabetes, uncontrolled
    - Congestive Heart Failure with exacerbations
    - COPD with exacerbations
    - Cancer with malnutrition or food insecurity
    - Asthma with exacerbations
    - High risk pregnancy
    - Developmental Disorder (e.g., Autism Spectrum Disorder)
    - Chronic kidney disease, Stage 4 or 5
    - Failure to Thrive / Malnutrition
    - Inflammatory bowel disease, uncontrolled

© 2023 McCurley JL et al. *JAMA Health Forum.*

3. Need for assistance with activities of daily living (ADL) or instrumental ADL (IADL)
4. High emergency department utilization (defined as 2 or more visits within six months, or 4 or more visits within a year)
5. High-risk pregnancy or pregnancy complications

In some cases, these criteria were adapted slightly for individual SSOs to ensure that the most appropriate patients for that service were enrolled.

Allowable Uses of Flexible Services Funding.
The following is a summary of the allowable uses of Flex with regards to (A) Good and Services and (B) Administrative Funding. This is not a comprehensive description of allowable uses.

Good and Services
Two domains of goods and services were supported by Flex: Tenancy Preservation Supports and Nutrition Sustaining Supports. Allowable uses for these two domains included:

Tenancy Preservation Supports.

1. *Pre-Tenancy Supports – Individual Supports*. This category included services such as assessing housing needs, assistance with housing applications, assistance with understanding tenants' rights, services to establish a safe and healthy living environment, and transportation to receive these services.
2. *Pre-Tenancy Supports – Transitional Assistance*. This category included services to assist the enrollee with one-time household set-up costs and move-in expenses.
3. *Tenancy Sustaining Supports*: This category included services such as (but not limited to):
    - Assisting the enrollee with communicating with a landlord or property manager about disability accommodations
    - Assisting the enrollee with obtaining and maintaining discretionary or entitlement benefits, including completing, filing, and monitoring housing applications.
    - Assisting the enrollee in all aspects of the tenancy, including legal advocacy and legal services
    - Assisting the enrollee with adaptive skills needed to function and live independently and safely in the community
    - Assisting or providing the enrollee with transportation to these services

4. *Home Modification.* Home modifications included limited physical adaptations to the enrollee's community-based dwelling when necessary to ensure the member's health, welfare, and safety, or to enable the member to function independently in a community-based setting.

Nutrition Sustaining Supports. This category included goods, transportation, and services to help enrollees access food needed to meet their nutritional needs, as well as nutrition education services. Examples of nutrition sustaining supports included:

- Assisting the enrollee with obtaining entitlement benefits (e.g., SNAP)
- Assisting the enrollee with access to foods that meet nutritional and dietary needs
- Providing the enrollee with nutrition education and skills development
- Providing the enrollee with healthy home-delivered meals
- Assisting the enrollee with transportation to any of these services

© 2023 McCurley JL et al. *JAMA Health Forum.*

Goods and Services for Children
For enrollees under the age of 19, a parent, guardian, or caregiver of the child could receive Flexible Services on the child's behalf when the following conditions were met:

- The delivery of the service to the parent, guardian, or caregiver was in the best interest of the child as determined by the ACO;
- Such determination was documented in the child's Flexible Services Plan; and
- The parent, guardian, or caregiver resided with the child.

Administrative Funding
ACOs could use Flexible Services funding to pay for the following:

- Delivery of services by SSO staff
- Administrative costs of SSOs delivering services
- Delivery of services by ACO staff if delivered internally (i.e., not through an SSO)
- Administrative costs of ACOs delivering services internally
- Costs of Flexible Services goods
- Salaries of ACO staff to oversee the administration of Flex*
- Costs of pre-delivery and post-delivery activities including identifying, screening, planning, navigating, collecting, and collating data
- Development of workflows and programs
- Overhead costs such as prorated costs of office space rent and utilities

*Funding of ACO staff time for program administration was not an allowable use at the start of the Flex program. MassHealth added this to the list of allowable uses in June of 2020, several months after the program had begun.

SSO-based administrative charges did not have a cap or limit. ACO administrative charges could not exceed an annual administrative cap of 15% of overall spending.

Target Number of Members Enrolled
MassHealth did not establish target enrollment numbers for each ACO. ACOs were allotted a maximum per member per month allowable budget based on their ACO enrollment. Each ACO developed a proposal for participation in Flexible Services with a target number of enrollments for their ACO based on their allowable budget. The MGB ACO planned for approximately 5,100 Flex enrollments from the two hospitals included in this study (Massachusetts General Hospital and Brigham and Women's Hospital) during the 3-year pilot. During the time period assessed in this study (March 2020-July 2021), the target enrollment for the two hospitals was 1900. As Flex enrollees could be re-enrolled for continues services, this number referred to the number of enrollments, not the number of individuals enrolled.

Performance / Evaluation Metrics
ACOs were required to report Flex enrollments to MassHealth quarterly. In addition, each ACO was required to submit annual reporting of an a priori-determined clinical measure, utilization measure, and process measure of their choosing for Flex participants.

For the MGB ACO, the clinical measures selected were health-need-specific (e.g., HbA1c for Flex enrollees with type 2 diabetes, BMI for Flex enrollees with obesity). The utilization measure selected was total cost of care, and the process measure was change in the Flex enrollee's qualifying social need (i.e., food or housing insecurity).

© 2023 McCurley JL et al. *JAMA Health Forum.*

Other social needs programming in the ACO
Separately from Flex, MassHealth ACOs were contractually required to screen ACO members for social needs, and screening rates were included in the slate of quality and performance metrics that comprised each ACO's evaluation for overall payment. Other DSRIP-funded programs in Massachusetts, such as Community Partners (partnerships between ACOs and community-based entities to provide behavioral health and long-term services and support) also targeted assessment and referrals for ACO members' social needs. Members' participation in these program may have increased the likelihood that they were referred to Flex. A unique feature of Flex compared to other similar programs was the direct payment for social needs services via funding contracts with community based SSOs.

Data sharing between ACO and SSOs
Each ACO was responsible for establishing their own process for data sharing between the ACO and community-based SSOs. The MGB ACO partnered with a pre-existing online social resource platform called "*findhelp*" (previously called "Aunt Bertha") at the start of Flex to integrate a communication and tracking platform that the MGB health system could use to coordinate Flexible Services with community based SSOs. When eligible ACO members were enrolled in Flex, referrals could be securely transmitted to SSOs using *findhelp* and a designated enrollment form. Modules were created within *findhelp* for SSOs to track service delivery outcomes for their Flex-enrolled clients. Utilization of *findhelp* for these purposes was not mandatory, and use of this platform by SSOs was inconsistent, particularly during the first year of Flex. Many SSOs utilized manual referral processes and/or existing internal client and service delivery databases.

References
[1] Commonwealth of Massachusetts. Massachusetts Delivery System Reform Incentive Payment Program. Accessed February 14, 2023. https://www.mass.gov/info-details/massachusetts-delivery-system-reform-incentive-payment-program.

© 2023 McCurley JL et al. *JAMA Health Forum.*

eTable. Health conditions documented by health center staff as eligibility criteria[2] for enrollment into Flexible Services for patients aligned to two large hospitals in the Mass General Brigham Accountable Care Organization from March 2020 - July 2021.

Health conditions[a] documented as Flex eligibility criteria[b]	Adult Flex enrollees (N = 658)		Pediatric Flex enrollees (N = 173)	
	N	%	N	%
Complex physical health conditions				
Obesity (for adults, BMI >30; for children, BMI >95th %)	428	65.1	103	59.5
Hypertension, uncontrolled[c]	102	15.5	0	0
Diabetes, uncontrolled[d]	90	13.7	2	1.2
Congestive Heart Failure with exacerbations	20	3.0	1	0.6
COPD with exacerbations	15	2.3	0	0
Cancer with malnutrition or food insecurity	13	2.0	2	1.2
Asthma with exacerbations	12	1.8	10	5.8
High risk pregnancy	11	1.7	0	0
Developmental Disorder (e.g., Autism Spectrum Disorder)	6	0.9	31	17.9
Chronic kidney disease, Stage 4 or 5	6	0.9	0	0
Failure to Thrive / Malnutrition	4	0.6	9	5.2
Inflammatory bowel disease, uncontrolled	2	0.3	0	0
Other conditions	126	19.2	61	35.3
Behavioral health condition				
Depression, uncontrolled[e]	142	21.6	7	4.1
Anxiety, uncontrolled[f]	85	12.9	8	4.6
Substance use disorder	49	7.5	0	0
Bipolar disorder	23	3.5	0	0
Schizophrenia	5	0.8	0	0
Other	52	7.9	21	12.1
High Emergency Department Utilization[g]	37	5.6	7	4.1
Both physical and mental health condition indicated	157	23.9	20	11.6

Flex=Flexible Services program; COPD=chronic obstructive pulmonary disease

[a] Health conditions documented to indicate eligibility for Flex, not a complete list of patients' medical diagnoses.
[b] Eligibility criteria for Flex included: (1) enrollment in MGB Medicaid ACO, (2) food or housing insecurity identified by screening or clinical encounter, and (3) either a complex health condition (e.g., uncontrolled diabetes, depression) or high ED utilization.
[c] Uncontrolled hypertension = hypertension diagnosis and systolic blood pressure > 140/90.
[d] Uncontrolled diabetes = for adults, type 2 diabetes diagnosis and hemoglobin A1c (HbA1c) > 8%; for children, type 1 diagnosis and HbA1c > 7.5% or type 2 diagnosis and HbA1c > 7%.
[e] Uncontrolled depression = Patient Health Questionnaire-9 score ≥ 10.
[f] Uncontrolled anxiety = Generalized Anxiety Disorder-7 score ≥ 10.
[g] High ED utilization defined as ≥ 2 visits in the last 6 months or ≥ 4 visits in the last 12 months.

© 2023 McCurley JL et al. *JAMA Health Forum.*

eFigure. Semi-structured interview scripts for qualitative interviews with health system staff and Flexible Services program enrollees.

SEMI-STRUCTURED INTERVIEW SCRIPT FOR STAFF

Role of Interviewee

1. What is the title of your job or position?
2. How long have you been working in this job?
3. What is your role with regards to the Flexible Services (FS) program? When did you start work on the FS program?

Program Objectives

4. What were the primary objectives for the FS program during the first year?
5. Were these objectives met? Why or why not? Did the objectives change over time?

Challenges

6. What have been the primary challenges to roll-out/launch of the FS program? (*probe each of the below areas if not mentioned)*

    - External factors
        - MassHealth/state policies
        - COVID-19
        - Other?
    - Internal factors
        - Central ACO factors
        - Differences between hospitals
        - Partnering with SSOs
        - Patient-related
        - Other?

7. What has facilitated roll-out, or helped things go better over time? *(probe each of the below areas if not mentioned)*

    - External factors
        - MassHealth/state policies
        - COVID-19
        - Other?
    - Internal challenges
        - Central ACO factors
        - Differences between hospitals
        - Partnering with SSOs
        - Patient-related
        - Other?

© 2023 McCurley JL et al. *JAMA Health Forum.*

Differences by Hospital (if applicable)

8. Can you describe the primary differences in the Flex launch in the first year by hospital?

Changes and Modifications

9. Have there been changes or modifications to the program, either by the state/MassHealth or locally, that have been important? *(probe each of the below areas if not mentioned)*

    - Any aspects of the program not being implemented as planned?
    - Modifications at any of these levels impacting implementation?
        - MassHealth/state
        - ACO
        - Hospitals/clinics

Similar Programs

10. How does the FS launch and implementation compare to previous, similar programs within the ACO? (with follow-up in each of the following areas)

SEMI-STRUCTURED INTERVIEW SCRIPT FOR FLEX ENROLLEES

1. As you know, we contacted you for this interview because you were enrolled by staff in your health center (*name health center*) in a program to receive resources or support related to food, nutrition, or housing in (*year*), or you were potentially eligible for these programs. Do you remember being signed up for a program like this? *(Prompt with names of community organizations if needed).*

2. Do you remember approximately when were you started the program, and about how long you participated in it?

3. What was your experience like getting signed up for this program? Was it easy for you? Was anything hard about it?

4. What type of resources or support did you receive?
    a. Which community organizations?
    b. Frequency of service/contact
    c. Were you satisfied with the resource or support service?

5. In what ways were these services helpful to you?
    a. Did you notice any changes in you or your family’s health?

6. Did these services help to solve the problem you had with food or housing?
    a. Why / why not?

7. Was anything difficult about accessing these services?
    a. Communication with the community organization
    b. Understanding the program/service
    c. Transportation to receive the service
    d. Time burden to access the service

© 2023 McCurley JL et al. *JAMA Health Forum.*

8. What was your experience like working with the community organization(s) that provided the services?
    a. Positive aspects
    b. Challenges/barriers

9. Was anyone at your health center involved in the process, for example in helping you communicate with the community organization(s)?
    a. How?
    b. Was this helpful?

10. Do you have recommendations for how this program should be changed or improved?

© 2023 McCurley JL et al. *JAMA Health Forum.*
