# Supplementary material for: Assessment of the Massachusetts Flexible Services Program to Address Food and Housing Insecurity in a Medicaid Accountable Care Organization
Source: JAMA Health Forum. 2023 Jun 2;4(6):e231191. doi: 10.1001/jamahealthforum.2023.1191 (PMC10238945; doi:10.1001/jamahealthforum.2023.1191)
Supplement: Supplement 2. — Data Sharing Statement [file jamahealthforum-e231191-s002.pdf]

Data Sharing Statement

McCurley. Assessment of the Massachusetts Flexible Services Program to Address Food and Housing Insecurity in a Medicaid Accountable Care Organization. *JAMA Health Forum*. Published June 02, 2023. doi:10.1001/jamahealthforum.2023.1191

Data
Data available: No
